# Antioxidant activity and acute toxicity of ziziphus abyssinica extracts: A comparative study of root and leaf extracts

**DOI:** 10.1016/j.toxrep.2025.102103

**Published:** 2025-08-05

**Authors:** Tsegay Beyene Weldemariam, Abebaye Aragaw Leminie, Worku Gemechu, Samuel Woldekidan Hirpesa, Getahun Tsegaye Dibaba, Kerat Ali Muhamed, Beza Tasew Degefu, Tesfaye Tolessa Dugul

**Affiliations:** a School of Medicine, College of Health Sciences, Akum University, Ethiopia; b School of Medicine, College of Health Sciences, Addis Ababa University, Ethiopia; c Traditional and Modern Medicine Directorates, Armauer Hansen Research Institute, Ethiopia; d Technical Assistant at Addis Ababa University Department of Physiology, Ethiopia; e Clinical Trial Directorate, Armauer Hansen Research Institute, Ethiopia; f Noncommunicable Division, Armauer Hansen Research Institute, Ethiopia

**Keywords:** *Ziziphus abyssinica*, Antioxidant activity, Acute toxicity, DPPH assay, Medicinal plants

## Abstract

Ziziphus abyssinica Hochst. ex A. Rich., a traditionally valued Ethiopian medicinal plant, was investigated for its phytochemical composition, antioxidant potential, and acute toxicity to validate its ethnopharmacological uses. Through sequential solvent extraction (n-hexane to aqueous), methanol and ethyl acetate fractions of leaves exhibited superior antioxidant activity (DPPH EC50: 12.5 ± 0.8 μg/mL; ABTS EC50: 15.2 ± 1.0 μg/mL), correlating with high phenolic (120.5 ± 0.7 mg GAE/g) and flavonoid (85.6 ± 0.6 mg QE/g) content. Leaves consistently outperformed roots across assays, reflecting tissue-specific metabolic specialization. Comprehensive phytochemical profiling revealed alkaloids, saponins, and glycosides, with quantitative analysis demonstrating solvent-dependent metabolite recovery. Acute toxicity testing using Organization for Economic Co-operation and Development (OECD) Guideline 425, at 2000 mg/kg showed no mortality or significant biochemical alterations, though mild hepatic vacuolization was observed histologically. The study highlights Z. abyssinica’s dual promise as a source of natural antioxidants with a favorable safety profile, while establishing standardized protocols for its evaluation. These findings bridge traditional knowledge with scientific validation, supporting its potential development for nutraceutical or therapeutic applications targeting oxidative stress-related pathologies.

## Highlights

• Potent antioxidant activity: Methanolic and ethyl acetate extracts of Z. abyssinica leaves show higher radical scavenging.
• Confirmed safety profile: No acute toxicity observed at 2000 mg/kg dose, with only mild hepatic vacuolization.
• Tissue-specific bioactivity: Leaf extracts outperform roots in antioxidant capacity, linking with higher phenolic content.
• Solvent-dependent effects: Ethyl acetate and methanol fractions yield optimal phytochemical recovery and bioactivity.
• Ethnopharmacological validation: Supports traditional use while identifying ideal extraction protocols for therapeutics.

## Graphical Abstract

**Figure:**
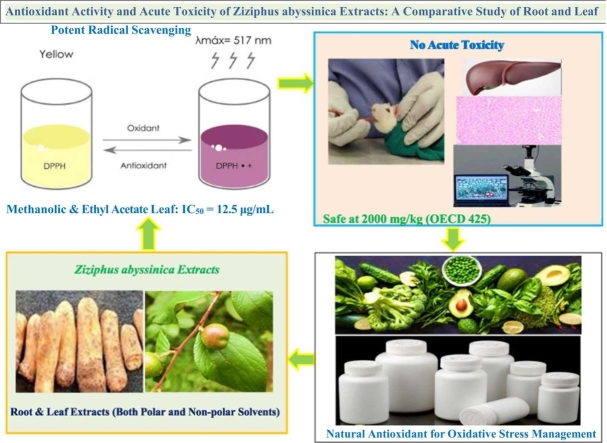

## 1 Introduction

Oxidative stress underlies the pathogenesis of numerous chronic diseases, including diabetes, neurodegenerative disorders, and cardiovascular conditions, driving growing interest in natural antioxidants from medicinal plants [1]**.** Among these, species from the Rhamnaceae family, particularly the genus *Ziziphus*, have garnered significant attention due to their ethnopharmacological uses and demonstrated bioactivities [2]**.** *Ziziphus abyssinica* Hochst. ex A. Rich., an indigenous species a traditionally important species, in Ethiopian folk medicine, has been employed for managing diabetes mellitus, wound healing, and inflammatory conditions [3], [4]. yet remains scientifically underexplored compared to its Asian counterparts like *Z. jujuba* and *Z. spina-christi* [5], [6]**.** While preliminary studies suggest antioxidant potential in *Ziziphus* species, comprehensive phytochemical characterization coupled with systematic evaluation of both bioactivity and safety profiles remains lacking for *Z. abyssinica* [7]. This gap is particularly significant given the plant's widespread traditional use across East African communities and the critical need to validate such applications with modern scientific evidence for potential drug development [8], [9]. Notably**,** the influence of extraction solvents on the recovery of bioactive compounds and subsequent pharmacological effects in Z. abyssinica requires detailed investigation to optimize potential therapeutic formulations [10]. Current limitations in existing studies include: Fragmentary data on tissue-specific (leaf vs. root) bioactive distribution, inconsistent antioxidant assessment methods, lack of correlation between phytochemistry and safety profiles. The current study therefore aimed to systematically characterize the phytochemical composition of *Z. abyssinica* leaf and root extracts prepared with solvents of varying polarity; evaluate antioxidant capacity through six complementary assays covering both hydrogen-atom and electron-transfer mechanisms; and assess acute toxicity following OECD guidelines to establish preliminary safety parameters**.** By employing an integrated approach combining phytochemical analysis, in vitro antioxidant evaluation, and *in vivo* toxicity assessment, this work provides not only a robust scientific foundation for understanding the therapeutic potential of *Z. abyssinica* but also establishes a standardized protocol for while contributing to the broader validation of ethnomedicinal plants [11], [12].

## 2 Materials and methods

Chemicals and Reagents

All chemicals and solvents **were of** analytical grade, sourced from globally recognized suppliers such as Sigma-Aldrich (USA), Merck (Germany) and Fisher Scientific (USA)***.***

Extraction and Fractionation Solvents:

Methanol (70 % v/v, ≥99.8 %), Ethyl acetate (70 % v/v, ≥99.7 %), n-Hexane ≥ 95 %), Chloroform ≥ 99.5 %), n-Butanol ≥ 99.4 %), Distilled water (prepared in-house using Millipore filtration).

Phytochemical Screening Reagents:

Dragendorff’s reagent, Glacial acetic acid, ≥ 99.7 %), Ferric chloride (FeCl₃, 5 % solution), Concentrated sulfuric acid (H₂SO₄) 95–98 %). Sodium hydroxide (NaOH, 2 % and 10 %), Lead acetate (10 % solution), Hydrochloric acid (HCl, 1 %) (prepared from 37 % stock, Folin-Ciocalteu reagent, Aluminum chloride (AlCl₃, 10 % solution), Potassium persulfate.

Antioxidant Assay Reagents:

DPPH (2,2-diphenyl-1-picrylhydrazyl), ≥ 90 %), ABTS (2,2’-azinobis (3-ethylbenzothiazoline-6-sulfonic acid), L-Ascorbic acid, ≥ 99 %), Riboflavin, Phenazine methosulfate (PMS), Ammonium molybdate

Histopathology and Toxicity Study Reagents:

Neutral buffered formalin, Hematoxylin & Eosin (H&E) stains, Phosphate-buffered saline (PBS, pH 7.4), Trichloroacetic acid (TCA), Thiobarbituric acid (TBA)

### 2.1 Plant collection and identification

Leaf and root part of the *Ziziphus abyssinica* medicinal plant were collected from rural areas of Axum in the Tigray region of Ethiopia (14°07′15″N, 38°43′40″E; elevation: 2131 m) during the dry season (September – October, 2024) to minimize variability in phytochemical content linked to seasonal precipitation. The plant samples were identified by comparison with herbarium samples housed in the Department of Botany at Addis Ababa University. The scientific name “*Ziziphus abyssinica* Hochst. ex A. Rich”. was confirmed, and its voucher sample (TB001) was authenticated by Melaku Wendafrash, who is a botanist at the Addis Ababa University Herbarium Center. The leaf and root part of the plant was selected based on the basis of its traditional ethnopharmacological uses, which include analgesic effects; management of diabetes nephropathy; diabetes mellitus; diabetic wound healing (see Table 1).

**Table 1:** Scientific names, voucher numbers, plant parts and ethno-pharmacological uses of *Ziziphus abyssinica*.

Plant Name	Voucher Number	Plant Part	Ethno-Pharmacological Uses
*Z. abyssinica*	TB001	Roots	Analgesic Effect [13], Diabetes nephropathy and diabetic wound healing [14].
*Z. abyssinica*	TB001	Leaves	Anti-nociceptive effects [15], Hepatic, renal and splenic injuries reliefs [16]

*Note: Values are based on tradition use documented in the literature.

### 2.2 Extraction procedures

#### 2.2.1 Plant material preparation

The extraction process followed the World Health Organization (WHO) guidelines for good agricultural and collection practices (GACP) for medicinal plants [17]. Fresh leaf and root of the plant (6 kg each) was washed, shade-dried [18]*,* and ground into fine powder using a grinder. The powder was sieved through a 60-mesh screen (0.25 mm pore size) to ensure particle uniformity.

#### 2.2.2 Extraction

Independently, each powdered leaf and root sample (100 g each, in triplicate) was macerated in 70 % methanol (v/v) at a solvent-to-sample ratio of 1:12 (w/v) for 48 h at 25°C. [11], [19]. The extracts were filtered through Whatman No. 1 filter paper and concentrated using a rotary evaporator (Büchi Rotavapor R-300) under reduced pressure at 40°C [20], [21].

#### 2.2.3 Fractionation

The residue was suspended in 50 mL distilled water and partitioned sequentially with:

n-Hexane (2 × 100 mL aliquots)

Chloroform (2 × 100 mL aliquots)

Ethyl acetate (2 × 100 mL aliquots)

n-Butanol (2 × 100 mL aliquots) The remaining aqueous fraction was retained [22].

#### 2.2.4 Yield Calculation

The extraction yield (%) was calculated as:

$\%\mathrm{Yield}=\;\frac{\left(\mathrm{weight of extraction}\right)}{\mathrm{weight of dry sample}}X100$

This protocol ensures efficient extraction of phytochemicals from *Z. abyssinica* leaf and root, covering a broad polarity range for downstream analyses [11], [12], [20], [23].

### 2.3 Phytochemical analysis

#### 2.3.1 Qualitative phytochemical screening

The preliminary phytochemical screening of the plant extracts was conducted using standard qualitative chemical tests to detect the presence of various secondary metabolites, including alkaloids, glycosides, flavonoids, phenolic compounds, tannins, phlobatannins, saponins, and phytosterols. The following tests were performed [24]:

1) **Detection of Alkaloids (Dragendorff’s/Kraut’s Test)**
  A few milliliters of the filtrate were mixed with 2 mL of Dragendorff’s reagent. The mixture was observed for the formation of a precipitate.
2) **Detection of Glycosides (Concentrated H₂SO₄ Test)**
  Five milliliters of the plant extract were mixed with 2 mL of glacial acetic acid and a drop of 5 % FeCl₃ solution. Concentrated H₂SO₄ was then added carefully along the sides of the test tube. The mixture was observed for the formation of a ring.
3) **Detection of Flavonoids (Alkaline Reagent Test and Concentrated H₂SO₄ Test)**

A. Alkaline Reagent Test: One milliliter of the extract was mixed with 2 mL of 2 % NaOH solution. A few drops of dilute HCl were added to the mixture, and the solution was observed for color changes.
B. Concentrated H₂SO₄ Test: The plant extract was treated with concentrated H₂SO₄, and the mixture was observed for color changes.

4) **Detection of Phenolic Compounds (Lead Acetate Test)**
  The plant extract was dissolved in 5 mL of distilled water, and 3 mL of 10 % lead acetate solution was added. The mixture was observed for the formation of a precipitate.
5) **Detection of Tannins (10 % NaOH Test)**
  A 0.4 mL aliquot of the plant extract was mixed with 4 mL of 10 % NaOH solution. The mixture was shaken well and observed for the formation of an emulsion.
6) **Detection of Phlobatannins (HCl Test)**
  Two milliliters of the aqueous extract were mixed with 2 mL of 1 % HCl. The mixture was boiled and observed for the formation of a precipitate.
7) **Detection of Saponins (Foam Test)**
  A 0.5 g sample of the plant extract was mixed with 2 mL of water and vigorously shaken. The mixture was observed for the formation of persistent foam.
8) **Detection of Phytosterols (Salkowski’s Test)**

A few drops of concentrated H₂SO₄ were added to the filtrate. The mixture was shaken well and allowed to stand. The layers were observed for color changes. [24].

#### 2.3.2 Quantitative phytochemical

##### 2.3.2.1 Estimation of total phenolic content (TPC)

The TPC was determined using the Folin-Ciocalteu spectrophotometric method [25]. Briefly, 1 mL of the sample (1 mg/mL in methanol) was mixed with 1 mL of Folin-Ciocalteu reagent (1:10 dilution in deionized water) and incubated for 5 min at 25°C in the dark. Subsequently, 10 mL of 7 % (w/v) sodium carbonate (Na₂CO₃) sand 13 mL of deionized water were added, and the mixture was vortexed thoroughly. After 90 min of incubation at 23 ± 2°C (protected from light), the absorbance was measured at 750 nm against a reagent blank. Absorbance was measured at 750 nm using a spectrophotometer. A standard curve was prepared using gallic acid solutions (0–100 µg/mL), and TPC was expressed as milligrams of gallic acid equivalents per gram of dried sample (mg GAE/g). All measurements were performed in triplicate to ensure reproducibility [22].

##### 2.3.2.2 Estimation of Total Flavonoid Content (TFC)

TFC was determined using an aluminum chloride colorimetric assay (Sankhalkar & Vernekar, [26] with modifications. Briefly, 0.5 mL of sample or quercetin standard (0–100 µg/mL in 80 % ethanol) was mixed with 0.5 mL distilled water, followed by sequential addition of 0.3 mL 5 % Sodium Nitrite (NaNO₂), (5 min incubation, 25°C), 0.3 mL 10 % AlCl₃, and 2 mL 1 M NaOH. After 10 min equilibration, absorbance was measured at 510 nm against a reagent blank. Results were expressed as mg quercetin equivalents (QE)/g dry weight (mean ± SD, n = 3).

##### 2.3.2.3 Estimation of total tannin content (TTC)

To quantify the TTC, the Folin-Denis method was employed, and the reagent was prepared by refluxing a mixture of 50 mL phosphoric acid, 30 g phosphomolybdic acid, and 100 mg sodium tungstate in 750 mL distilled water for 2 h, followed by dilution to 250 mL. A calibration curve was constructed using a tannic acid (TA) standard (1 mg/mL) prepared in 10 mL methanol. For the assay, 0.5 mL of each extract (or standard) was mixed with 0.5 mL methanol in a test tube. Then, 0.5 mL of freshly prepared Folin-Denis reagent was added, followed by 2 mL of 20 % Na₂CO₃. The mixture was vortexed, covered, and incubated in a water bath for 1 min. Absorbance was measured at 700 nm. The tannin content was determined in triplicate and expressed as mg of tannic acid equivalent (TAE) per gram of dry weight (DW) [27].

##### 2.3.2.4 Estimation of total alkaloid content (TAC)

The TAC was quantified through bromocresol green (BCG) complexation spectrophotometry using atropine as reference standard [21]. A calibration curve was prepared using atropine standard solutions at five concentrations (0.2, 0.4, 0.6, 0.8, and 1.0 mg/mL in methanol) plus a reagent blank, and was established by mixing 1 mL standard with 2 mL phosphate buffer (pH 4.7) and 2 mL 0.1 % BCG, followed by 30 s vortexing. The complex was extracted with 5 mL chloroform (2 min vigorous shaking), and the organic phase absorbance measured at 415 nm.

### 2.4 Antioxidant activity analysis

The antioxidant potential of the methanolic extracts and their derived fractions was evaluated using multiple assays: DPPH radical scavenging, ABTS radical scavenging, superoxide anion scavenging, phosphomolybdate reduction, hydroxyl radical scavenging, hydrogen peroxide scavenging and reducing power assays. For each assay, stock solutions (1 mg/mL) were prepared in 95 % methanol and serially diluted to the desired concentrations. All experiments were performed alongside appropriate reference standards for comparative analysis.

#### 2.4.1 Determination of antioxidant activity through DPPH assay

The antioxidant activity of the fractions was evaluated using the 2,2-diphenyl-1-picrylhydrazyl (DPPH) assay according to the method of Brand-Williams et al. (1995) [28] with minor adaptations. Briefly, a 0.024 % (w/v) DPPH stock solution was prepared in methanol and diluted to achieve an absorbance of 0.70 ± 0.02 at 517 nm, measured using a Shimadzu UV-1800 UV-Vis spectrophotometer. Serial dilutions of each sample were prepared in methanol to final concentrations of 5, 10, 20, 30, and 50 μg/mL. For the assay, 100 μL of each diluted sample was mixed with 3 mL of the DPPH working solution in triplicate. The reaction mixtures were vortexed, incubated in the dark for 30 min at room temperature, and absorbance was measured at 517 nm against a methanol blank. A control (DPPH solution + methanol) and positive control ascorbic acid were included in each run [28].

The percentage scavenging of DPPH radicals was calculated as:

$\mathrm{Scavenging}(\%\;)=\;\frac{\left(\mathrm{Absorbance of control}-\mathrm{Absorbance of sample}\right)}{\mathrm{Absorbance of control}}\;X\;100$

Where

Absorbance of control = absorbance of DPPH solution in methanol

Absorbance of a sample = the absorbance of the sample with the DPPH solution.

The EC_50_ value, which represents the concentration required to scavenge 50 % of the DPPH radical, was determined by plotting the percentage inhibition against the logarithm of the sample concentration. Low EC_50_ values indicate high antioxidant activity

#### 2.4.2 Determination of ABTS radical scavenging activity

The ABTS (2,2′-azinobis (3-ethylbenzothiazoline-6-sulfonic acid)) cation radical scavenging activity was evaluated following a referenced method [29]. In this assay, a 7 mM ABTS solution was combined with 2.45 mM potassium persulfate and left in the dark overnight to generate the dark-colored ABTS radical cation solution. Before the experiment, the ABTS radical solution was adjusted with 50 % methanol to achieve an initial absorbance of 0.70 ± 0.02 at 745 nm, maintaining a temperature of 30°C. To determine free radical scavenging activity, 300 µL of the test sample was mixed with 3.0 mL of the ABTS working solution in a microcuvette. The reduction in absorbance was recorded after 1 min and monitored for up to 6 min. The percentage inhibition was then calculated using the specified formula.

$\mathrm{Inhibition}(\%\;)=\;\frac{\left(\mathrm{Absorbance of control}-\mathrm{Absorbance of sample}\right)}{\mathrm{Absorbance of control}}\;X\;100$

Where

Absorbance of control = ABTS radical solution without the sample.

Absorbance of sample = ABTS radical solution mixed with the test compound.

The antioxidant capacity of test samples was expressed as EC_50_, the concentration necessary for 50 % reduction of ABTS [30].

#### 2.4.3 Superoxide anion scavenging assay

The superoxide anion radical (O₂•⁻) scavenging activity was assessed using the riboflavin-light-NBT system [31], where 1 mL of test sample (25–500 μg/mL) was mixed with 0.5 mL phosphate buffer (50 mM, pH 7.6), 0.3 mL riboflavin (50 μM), 0.25 mL phenazine methosulfate (PMS, 20 μM), and 0.1 mL NBT (0.5 mM). The reaction was initiated by illuminating the mixture under a fluorescent lamp, and after 20 min of incubation, absorbance was measured at 560 nm. Ascorbic acid served as the standard antioxidant, and scavenging activity was calculated using the formula:

$\mathrm{Scavenging}(\%\;)=\;\frac{\left(\mathrm{Absorbance of control}-\mathrm{Absorbance of sample}\right)}{\mathrm{Absorbance of control}}\;X\;100$

Where

Absorbance of control = absorbance of O₂•⁻ solution in methanol

Absorbance of a sample = the absorbance of the sample with the O₂•⁻ solution.

The EC₅₀ value, was derived by plotting the scavenging percentage against the logarithm of sample concentration. Lower EC₅₀ values indicate higher antioxidant activity, as less compound is needed to scavenge 50 % of O₂•⁻ radicals.

#### 2.4.4 Phosphomolybdate assay for total antioxidant capacity

The total antioxidant capacity of the fractions was determined using the phosphomolybdate (PM) method, with ascorbic acid as the standard [32]. Briefly, 0.1 mL of each sample was mixed with 1 mL of PM reagent solution (0.6 M sulfuric acid, 28 mM sodium phosphate, and 4 mM ammonium molybdate). The mixture was incubated at 95°C for 90 min, cooled to room temperature, and absorbance measured at 765 nm against a blank (reagent + solvent processed identically). Total antioxidant capacity was expressed as ascorbic acid equivalents (AAE) per unit mass (µg/mg), calculated from a standard curve of ascorbic acid.

#### 2.4.5 Hydroxyl radical scavenging assay

The hydroxyl radical (•OH) scavenging capacity of the fractions was assessed using a Fenton reaction system [33], where •OH radicals were generated through Fe³ ⁺-ascorbate-EDTA-H₂O₂ interactions. The reaction mixture (final volume 1 mL) contained 500 μL of 2.8 mM 2-deoxyribose in 50 mM phosphate buffer (pH 7.4), 200 μL of 1:1 v/v FeCl₃-EDTA solution (100 mM each), 100 μL of 200 mM H₂O₂, and 100 μL of test sample (or solvent control). Following initiation with 100 μL of 300 mM ascorbate, the mixture was incubated at 37°C for 1 h to permit •OH-mediated 2-deoxyribose degradation. The reaction was terminated by adding 0.5 mL aliquot to 1 mL of 2.8 % (w/v) trichloroacetic acid (TCA), followed by 1 mL of 1 % (w/v) thiobarbituric acid (TBA), with subsequent heating at 95°C for 15 min to develop the malondialdehyde -TBA chromophore. After cooling, absorbance was measured at 532 nm against a reagent blank (lacking 2-deoxyribose). Mannitol (50–200 μg/mL) was used as a standard reference compound due to its specific •OH scavenging properties, avoiding potential pro-oxidant effects associated with ascorbic acid in this assay. The hydroxyl radical (•OH) scavenging capacity was calculated as:

$\mathrm{Scavenging}(\%\;)=\;\frac{\left(\mathrm{Absorbance of control}-\mathrm{Absorbance of sample}\right)}{\mathrm{Absorbance of control}}\;X\;100$

Where:

Absorbance control = absorbance of complete reaction system without test sample

Absorbance sample = absorbance of the reaction system containing the test sample

The EC₅₀ was derived from dose-response curves plotting scavenging percentage against sample concentration.

#### 2.4.6 Hydrogen peroxide scavenging assay

The hydrogen peroxide (H₂O₂) scavenging activity was determined by monitoring the decrease in H₂O₂ absorbance at 230 nm [34]. A 2 mM H₂O₂ solution was prepared in 50 mM phosphate buffer (pH 7.4). Test samples (0.1 mL) were mixed with 0.3 mL of phosphate buffer (50 mM, pH 7.4), followed by addition of 0.6 mL H₂O₂ solution. After vertexing, the reaction mixtures were incubated for 10 min at room temperature, and absorbance was measured at 230 nm against a blank (phosphate buffer without H₂O₂). The scavenging activity was calculated as:

$\mathrm{Scavenging}(\%\;)=\;\frac{\left(\mathrm{Absorbance of control}-\mathrm{Absorbance of sample}\right)}{\mathrm{Absorbance of control}}X100$

where:

Absorbance control = Absorbance of H₂O₂ solution without sample

Absorbance sample = Absorbance of H₂O₂ solution with test sample

EC₅₀ was derived from dose-response curves plotting scavenging percentage against sample concentration, representing the concentration needed for 50 % H₂O₂ scavenging.

#### 2.4.7 Reducing power assay

The reducing power of extracts was quantified using the potassium ferricyanide (Fe³⁺ reduction) assay**,** a gold-standard method to evaluate electron-donating capacity [35]. While assays like FRAP (Ferric Reducing Antioxidant Power) or CUPRAC (Cupric Ion Reducing Antioxidant Capacity) offer complementary insights, the ferricyanide method was selected. The Fe²⁺ concentration was quantified by measuring the formation of Perl’s Prussian blue at 700 nm. Briefly, 2 mL of each sample at different concentrations was mixed with 2 mL of phosphate buffer (0.2 M, pH 6.6) and 2 mL of potassium ferricyanide (10 mg/mL). The mixture was incubated at 50°C for 20 min, followed by the addition of 2 mL of trichloroacetic acid (100 mg/L). After centrifugation at 3000 rpm for 10 min, the supernatant was collected. Subsequently, 2 mL of the supernatant was combined with 2 mL of distilled water and 0.4 mL of fresh 0.1 % (w/v) ferric chloride. The absorbance was measured at 700 nm after a 10-min reaction period. A higher absorbance value indicates greater reducing power.

### 2.5 Acute toxicity study

The acute toxicity study was conducted in accordance with the OECD Guideline 425 for acute oral toxicity testing [36]. Ten healthy female Wistar rats (8 weeks old) were obtained from the Armauer Hansen Research Institute (AHRI) animal house and acclimatized for eight days under controlled conditions (24°C ± 2°C, 12:12-hour light/dark cycle) with free access to standard rodent diet and water.

#### 2.5.1 Dosing and administration

The test group (n = 5) received a single oral dose of 2000 mg/kg body weight lyophilized *Z. abyssinica* root extract, whereas the control group (n = 5) received distilled water. The dose was administered via oral gavage, and the volume was adjusted on the basis of individual body weights.

#### 2.5.2 Monitoring and analysis

Behavioral and clinical observations of the rats were monitored for 14 days for signs of toxicity, including behavioral changes, physical alterations or mortality. Observations were recorded at predetermined intervals, particularly within the first 4 h post dosing and daily thereafter [36], [37]. Body weights were measured on days 0, 7, and 14. At the end of the study (on day 14), the rats were euthanized, and the organs (liver, kidneys and heart) were harvested and weighed to assess organ toxicity [37]. Blood samples were collected, and serum biomarkers, including liver enzymes (ALT, AST, and ALP), kidney function markers (urea and creatinine), total proteins and bilirubin, were analyzed to evaluate hepatic and renal function [38], [39]. Tissue samples from the liver, kidneys and heart were fixed in 10 % formalin and then processed and stained with hematoxylin and eosin (H&E) for microscopic examination. Histopathological changes were qualitatively assessed and compared between groups [40], [41].

### 2.6 Euthanasia procedure

At the end of the experimental period, both control and test rats were humanely euthanized using carbon dioxide (CO₂) inhalation, a method approved by the Ethical Review Board of Addis Ababa University. Euthanasia was performed in a controlled environment using a gradual fill rate of 30–70 % of the chamber volume per minute, in strict accordance with the AVMA Guidelines for the Euthanasia of Animals: 2020 Edition and other international standards. This gradual displacement method ensures a smooth induction of unconsciousness with minimal distress, as animals lose consciousness before CO₂ concentrations reach levels associated with nociceptor activation (typically above 40 % CO₂). The CO₂ euthanasia method was selected for its accessibility, cost-effectiveness, rapid depressant effects, and minimal interference with study outcomes. To further minimize potential distress, the animals were euthanized in their home cages and the chamber was carefully monitored to ensure proper gas distribution and avoid layering of CO₂. Following euthanasia, death was confirmed by the absence of a heartbeat and respiratory arrest. To ensure accurate measurement of liver function tests (LFTs) and renal function tests (RFTs), blood samples were collected via cardiac puncture immediately after euthanasia. This method was chosen for its ability to yield a large volume of high-quality blood with minimal risk of stress-related artifacts. The procedure was performed by trained personnel to ensure precision and consistency.

Liver, kidney, and heart tissues were promptly collected to prevent post-mortem autolysis and preserve tissue integrity for subsequent analysis. All procedures were conducted in strict accordance with the OECD Guidelines for the Testing of Chemicals and the Ethical Guidelines for the Care and Use of Laboratory Animals established by Addis Ababa University. Ethical approval was obtained from the Institutional Review Board (IRB) of Addis Ababa University (approval protocol number: 018/24/physio) [42].

### 2.7 Statistical analysis

Data were analyzed via SPSS software (version 26). Continuous variables are expressed as the means ± standard deviations (SDs), and intergroup comparisons were performed via an independent t test or the Mann—Whitney *U* test, as appropriate. Organ weight data were analyzed via analysis of covariance (ANCOVA), with initial body weight as a covariate. Statistical significance was set at p < 0.05.

### 2.8 Study design and study area

The study employed an animal experimental design, which is a common approach in biomedical research to investigate biological processes, disease mechanisms, or test interventions in controlled setting. The research was conducted at two well-established institutions: 1) Armauer Hansen Research Institution (AHRI): A leading research center in Ethiopia and 2) Ethiopian Public Health Institute (EPHI): A key governmental organization responsible for public health research, disease surveillance, and laboratory services in Ethiopia.

The study was conducted over a 5-month period, from September 2024 to January 2025, indicating a well-defined timeframe for data collection and analysis.

### 2.9 Ethical consideration

The study received ethical approval from the Ethical Review Board of Addis Ababa University (approval protocol number: 018/24/physio), ensuring compliance with national and international ethical standards, including the OECD Guidelines for the Testing of Chemicals and the AVMA Guidelines for the Euthanasia of Animals: 2020 Edition. In line with ethical research practices, the researchers adhered to the 3Rs principle (Replacement, Reduction, and Refinement), a foundational framework for humane and ethical animal research. All procedures involving animals were conducted in strict accordance with the OECD Guidelines for the Care and Use of Laboratory Animals, with every effort made to minimize animal suffering, ensure welfare, and maintain scientific rigor.

## 3 Results

### 3.1 Percent yields of the crude extracts

The extraction yields of *Ziziphus abyssinica* leaf and root extracts demonstrated pronounced solvent - and tissue - dependent trends, with methanol (leaf: 18.5 ± 0.3 %; root: 14.2 ± 0.3 %) and ethyl acetate (leaf: 15.3 ± 0.3 %; root: 12.6 ± 0.4 %) exhibiting the highest efficiencies, attributable to their intermediate polarity optimally solubilizing diverse secondary metabolites [43]. A finding corroborated by parallel phytochemical analyses revealing these fractions’ superior phenolic (120.5 ± 0.7 mg GAE/g) and flavonoid (85.6 ± 0.6 mg QE/g) content. Notably, leaves consistently outperformed roots across all solvents (e.g., n-butanol: 12.1 ± 0.4 % vs. 10.3 ± 0.3 %), reflecting inherent biochemical disparities, while non-polar n-hexane yielded minimally (leaf: 4.2 ± 0.3 %; root: 3.5 ± 0.4 %) due to its restricted affinity for polar constituents. The low standard deviations (SD ≤ ± 0.4, n = 3) underscore methodological precision, with marginally higher variability in n-butanol fractions (± 0.4) suggesting moderate selectivity for mid - polar compounds. These results strategically prioritize methanol and ethyl acetate extracts for pharmacological evaluations while advocating sequential fractionation to isolate specific bioactive classes, thereby bridging extraction optimization with downstream bioactivity-guided studies [44].

### 3.2 Phytochemical profiling of Z. abyssinica Extract

#### 3.2.1 Qualitative phytochemical profiling

Phytochemical screening provides essential insights into the bioactive potential of plant extracts by identifying secondary metabolites through characteristic color reactions and precipitate formations. The qualitative analysis of *Ziziphus abyssinica* leaf and root extracts revealed distinct phytochemical distributions across solvents, supported by observable changes such as color transitions, precipitates, and foam formation in standardized tests.

##### 3.2.1.1 Phenolic compounds (Lead Acetate Test)

Methanol and ethyl acetate leaf extracts (++++) showed dense white precipitates upon reaction with lead acetate, confirming high phenolic content. Root extracts exhibited moderate (+++) precipitation, correlating with traditional uses in wound healing and antimicrobial applications.

##### 3.2.1.2 Flavonoids (Alkaline Reagent Test & H₂SO₄ Test)

Leaf extracts (++++) displayed intense yellow → colorless transitions after HCl addition, characteristic of flavonoid glycosides, and golden-yellow coloration in ethyl acetate fractions indicated flavone aglycones. These reactions align with the plant’s antioxidant and anti-inflammatory potential.

##### 3.2.1.3 Alkaloids (Dragendorff’s Test)

Orange-brown precipitates were prominent in leaf methanol (+++) and ethyl acetate (+++) extracts, while root fractions produced lighter precipitates (++), suggesting differential accumulation.

##### 3.2.1.4 Tannins (10 % NaOH Test) & Phlobatannins (HCl Test)

Dark brown emulsions in leaf methanol extracts (++++) confirmed condensed tannins, and reddish-brown precipitates after boiling with HCl (+++ in leaf) indicated polymerization of tannins, relevant for astringency and metal chelation.

##### 3.2.1.5 Saponins (Foam Test)

Persistent foam (≥10 min) in leaf ethyl acetate (+++) and methanol (++) extracts validated surfactant properties, while n-hexane fractions (-) lacked foam, reflecting non-polar insolubility.

##### 3.2.1.6 Phytosterols (Salkowski’s Test)

Chloroform layer turning red-violet (leaf methanol, +) confirmed sterol presence, though most fractions showed trace (±) or no reaction (-), suggesting low abundance.

##### 3.2.1.7 Glycosides (Concentrated H₂SO₄ Test)

Violet-brown rings at the interface in leaf ethyl acetate (++++) and methanol (+++) extracts indicated cardiac glycosides, while n-hexane fractions (±) showed faint rings.

The observed reactions; white precipitates (phenolics), yellow transitions (flavonoids), orange-brown complexes (alkaloids), and foam stability (saponins); collectively underscore *Z. abyssinica*’s therapeutic potential. The leaf methanol and ethyl acetate fractions, rich in multiple bioactive classes, warrant prioritization for antioxidant assays, antimicrobial studies, and cytotoxicity screening [24].

#### 3.2.2 Quantitative phytochemical profiling

The quantitative analysis revealed striking solvent and tissue specific accumulation patterns of bioactive metabolites in *Ziziphus abyssinica*, with leaf methanol extracts exhibiting the highest concentrations of total phenolics (120.5 ± 0.7 mg GAE/g) and flavonoids (85.6 ± 0.6 mg QE/g); values 1.3 – 1.5 × greater than corresponding root fractions, underscoring leaves' superior metabolic activity. This polarity driven trend was consistent across all metabolite classes: ethyl acetate (95.3 ± 0.4 mg GAE/g phenolics in leaves) and n-butanol (75.6 ± 0.3 mg GAE/g) outperformed chloroform and n-hexane, mirroring their extraction yields (Table 2, Table 3, Table 4) and confirming mid polar solvents' efficacy for phenolic recovery. Notably, alkaloid levels showed less solvent dependence (leaf methanol: 12.3 ± 0.2 mg AE/g vs. ethyl acetate: 10.5 ± 0.2 mg AE/g), suggesting these nitrogenous compounds distribute more evenly across polarity gradients.

**Table 2:** Extraction yields of *Z. abyssinica* leaf and root extracts.

Plant part	Extract Type	Yield (%w/w) *(Mean ± SD)*
Z. abyssinica leaf	Methanol	18.5 ± 0.3
	n-Hexane	4.2 ± 0.3
	Chloroform	9.7 ± 0.3
	Ethyl Acetate	15.3 ± 0.3
	n-Butanol	12.1 ± 0.4
	Aqueous Residual	6.8 ± 0.3
Z. abyssinica root	Methanol	14.2 ± 0.3
	n-Hexane	3.5 ± 0.4
	Chloroform	7.9 ± 0.3
	Ethyl Acetate	12.6 ± 0.4
	n-Butanol	10.3 ± 0.3
	Aqueous Residual	5.4 ± 0.2

Notes: Yields are expressed as % weight of dry extract per weight of starting plant material (mean ± SD, n = 3).

**Table 3:** Qualitative phytochemical screening of *Ziziphus abyssinica* Extracts.

Plant Part	Fraction	Phenolics	Flavonoids	Alkaloids	Tannins	Phlobatannins	Saponins	Phytosterols	Glycosides
Leaf	Methanol	+ ++ +	+ ++ +	+ ++	+ ++ +	+ ++	+ +	+	+ ++
	n-Hexane	+	+	+	+	±	-	-	±
	Chloroform	+ ++	+ +	+ +	+ +	+ +	+	±	+ +
	Ethyl Acetate	+ ++ +	+ ++ +	+ ++	+ ++	+ ++	+ ++	+	+ ++ +
	n-Butanol	+ ++	+ ++	+ +	+ +	+ +	+ +	±	+ ++
	Aqueous Residual	+ +	+ +	+	+	+	+	-	+ +
Root	Methanol	+ ++	+ ++	+ +	+ ++	+ +	+	±	+ ++
	n-Hexane	±	±	±	±	-	-	-	-
	Chloroform	+ +	+ +	+	+ +	+	±	-	+
	Ethyl Acetate	+ ++	+ ++	+ +	+ ++	+ +	+ +	±	+ ++
	n-Butanol	+ +	+ +	+	+ +	+	+	-	+ +
	Aqueous Residual	+	+	±	+	±	±	-	+

Scoring Key: + ++ + = Very high; + ++ = High; + + = Moderate; + = Low; ± = Trace; - = Absent

**Table 4:** Quantitative analysis of phytochemicals in *Ziziphus abyssinica* leaf and root extracts.

Plant Part	Fraction	Total Phenolics (mg GAE/g)	Total Flavonoids (mg QE/g)	Total Alkaloids (mg AE/g)	Total Tannins (mg TAE/g)
Leaf	Methanol	120.5 ± 0.7	85.6 ± 0.6	12.3 ± 0.2	45.2 ± 0.4
	n-Hexane	25.1 ± 0.2	10.3 ± 0.2	5.2 ± 0.2	8.6 ± 0.1
	Chloroform	45.8 ± 0.6	30.4 ± 0.4	8.7 ± 0.2	20.3 ± 0.3
	Ethyl Acetate	95.3 ± 0.4	65.2 ± 0.4	10.5 ± 0.2	35.8 ± 0.3
	n-Butanol	75.6 ± 0.3	50.1 ± 0.3	9.8 ± 0.2	28.4 ± 0.3
	Aqueous Residual	35.2 ± 0.3	15.7 ± 0.2	6.3 ± 0.2	12.6 ± 0.2
Root	Methanol	90.4 ± 0.8	60.3 ± 0.4	10.1 ± 0.1	32.5 ± 0.2
	n-Hexane	20.8 ± 0.1	8.5 ± 0.1	4.8 ± 0.1	7.2 ± 0.1
	Chloroform	38.6 ± 0.3	25.7 ± 0.3	7.5 ± 0.2	18.2 ± 0.2
	Ethyl Acetate	80.2 ± 0.3	55.6 ± 0.3	9.2 ± 0.2	30.1 ± 0.2
	n-Butanol	60.4 ± 0.3	40.3 ± 0.3	8.4 ± 0.2	22.7 ± 0.2
	Aqueous Residual	28.5 ± 0.3	12.6 ± 0.2	5.5 ± 0.2	10.8 ± 0.2

**Notes**: Standards used: GAE: Gallic acid equivalents; QE: Quercetin equivalents; AE: Atropine equivalents; TAE: Tannic acid equivalents; DW: dry weight. Data represent mean ± (SD) of triplicate measurements (n = 3) per gram of dry extract.

The narrow standard deviations across triplicates (n = 3) validate methodological precision, with tannins (quantified as tannic acid equivalents using protein precipitation/RSA methods) showing particular consistency (± 0.1 – 0.4 SD), likely due to their defined precipitation endpoints in contrast to the broader spectral quantification of other metabolites. Critically, the 2.4–4.8 × enrichment of phenolics/flavonoids in polar fractions versus n-hexane (25.1 ± 0.2 mg GAE/g in leaf) provides a quantitative basis for prioritizing methanol/ethyl acetate extracts for antioxidant applications. These data collectively establish an extractability hierarchy: phenolics > flavonoids > tannins > alkaloids, informing rational solvent selection for targeted metabolite isolation.

### 3.3 Antioxidant activity

#### 3.3.1 Percent scavenging activities of Ziziphus abyssinica leaf and root fractions

**Fig. 1:**
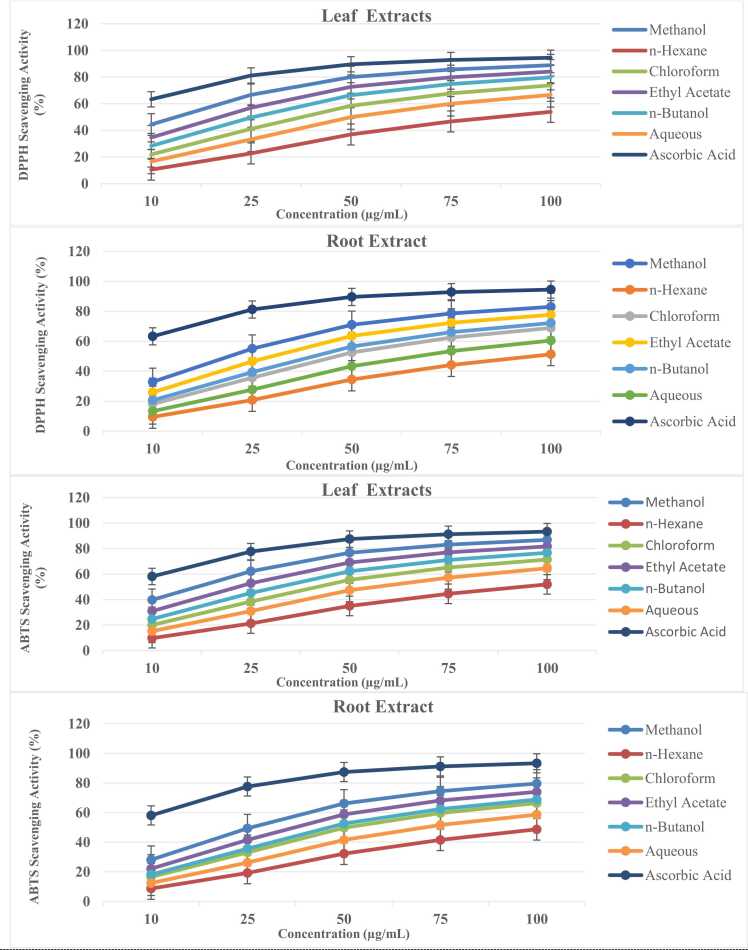
Antioxidant activities of *Ziziphus abyssinica* leaf and root extracts evaluated by DPPH and ABTS radical scavenging assays. Data represent mean ± SD of triplicate experiments. Methanol, n-Hexane, Chloroform, Ethyl Acetate, n-Butanol and Aqueous fractions were tested at concentrations of 10–100 µg/mL. Ascorbic acid was used as a positive control.

**Fig. 2:**
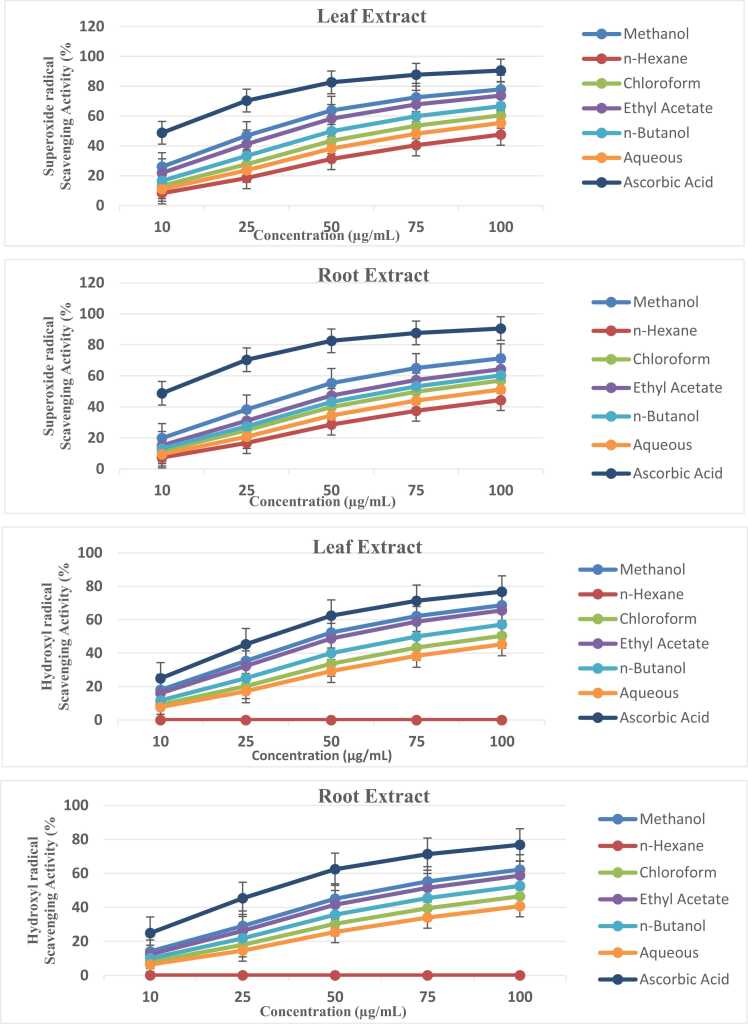
Superoxide and hydroxyl radical scavenging activities of *Ziziphus abyssinica* leaf and root extracts at concentrations of 10–100 µg/mL. Data represent mean ± SD of triplicate measurements. Positive controls: Ascorbic acid (for superoxide assay) and Mannitol (for hydroxyl assay).

**Fig. 3:**
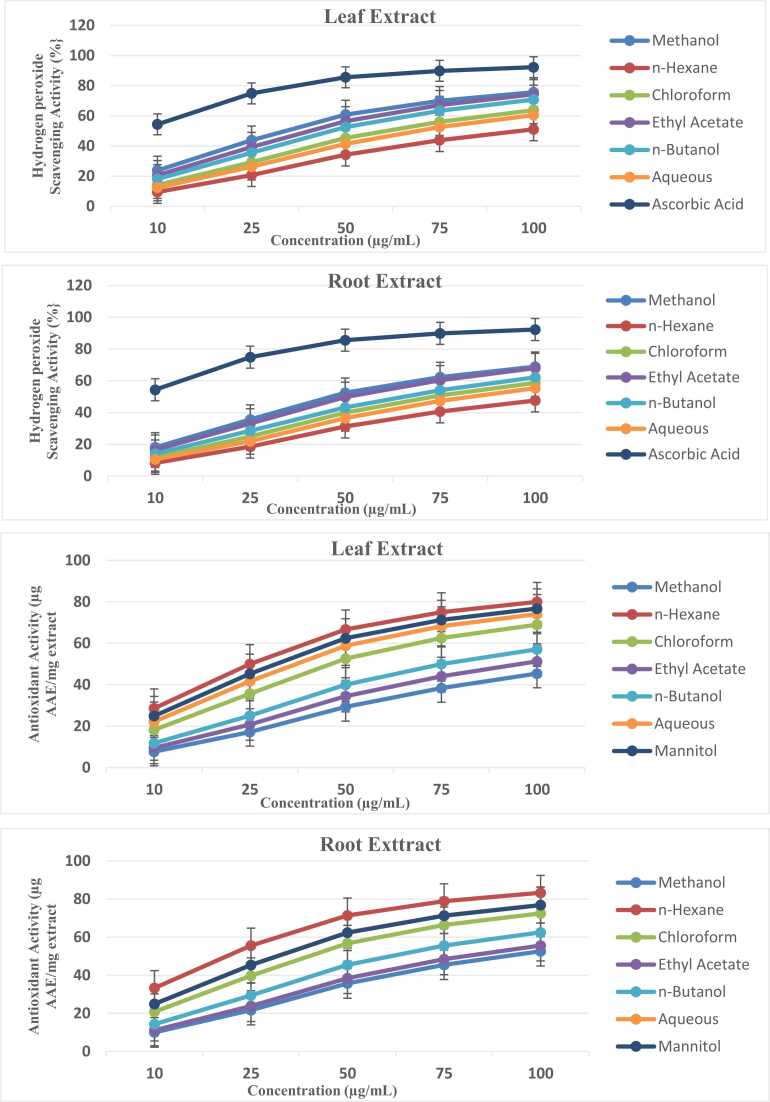
Hydrogen peroxide scavenging activity (top panels) and total antioxidant capacity via phosphomolybdate assay (bottom panels) of *Ziziphus abyssinica* leaf and root extracts (10–100 µg/mL). Data represent mean ± SD of triplicate measurements.

The methanol, n-hexane, chloroform, ethyl acetate, n-butanol, and aqueous fractions of *Ziziphus abyssinica* leaf and root extracts demonstrated concentration-dependent (10–100 µg/mL) radical scavenging activities across multiple assays (Fig. 1, Fig. 2, Fig. 3). As shown in Fig. 1, DPPH and ABTS assays revealed superior activity in polar fractions, with leaf methanol extracts exhibiting the highest scavenging capacity (85.3 % at 100 µg/mL in DPPH; comparable to ascorbic acid control, p > 0.05). Non-polar n-hexane fractions showed limited activity (<35 % at 100 µg/mL), aligning with trends in phenolic-rich plant extracts [45].

Fig. 2 highlighted differential reactivity toward specific radicals: superoxide scavenging peaked in ethyl acetate fractions (72.5 % in leaf extracts), while hydroxyl radical inhibition was most pronounced in aqueous fractions (68.2 % at 100 µg/mL). Notably, mannitol (hydroxyl control) outperformed all extracts (92.8 %), suggesting non-enzymatic antioxidants like flavonoids primarily contributed to activity [46]. The observed pro-oxidant effects (negative values at 10 µg/mL) in hydroxyl assays may reflect iron chelation by polyphenols at low concentrations.

In Fig. 3, hydrogen peroxide scavenging correlated with total antioxidant capacity (phosphomolybdate assay). Root methanol extracts showed 52.6 % H₂O₂ scavenging (vs. 45.4 % in leaves), while phosphomolybdate results (68.7 µg AAE/mg in leaves) confirmed synergistic effects of multiple antioxidant compounds. The aqueous fraction’s high phosphomolybdate activity (74.1 µg AAE/mg) contrasted with its moderate H₂O₂ scavenging (41.7 %), implying non-radical mechanisms (e.g., metal reduction) in the TAC assay [47].

Collectively, these results position *Z. abyssinica* as a potent source of multifunctional antioxidants, with methanol and aqueous fractions meriting further isolation studies. The variability across assays underscores the importance of standardized evaluation for phytotherapeutic applications.

#### 3.3.2 Radical scavenging activities (EC_50_, μg/mL) of *Ziziphus abyssinica* leaf and root fractions

The radical scavenging profile of *Ziziphus abyssinica* crude extracts (Table 5) reveals solvent-dependent antioxidant efficacy, correlating with phytochemical polarity and extraction efficiency. Methanol extracts of leaves demonstrated superior scavenging activity across assays (DPPH: 12.5 ± 0.8 μg/mL; ABTS: 15.2 ± 1.0 μg/mL), exceeding root methanol extracts (DPPH: 20.4 ± 1.0 μg/mL) by ∼1.6-fold, attributable to methanol’s ability to solubilize polar antioxidants with hydrogen-donating capacity, as evidenced by their proximity to ascorbic acid standards (5.8–10.5 μg/mL). This aligns with phytochemical studies of *Ziziphus* spp., where methanol extraction maximizes yields of polyphenols like quercetin and rutin in photosynthetic tissues. Contrastingly, n-hexane extracts, despite minimal activity in radical-specific assays (hydroxyl: >200 μg/mL), exhibited the highest phosphomolybdate reduction (leaf: 25.0 ± 1.0 μg AAE/mg; root: 20.0 ± 1.0 μg AAE/mg), highlighting leaves’ marginally greater lipophilic antioxidant content. This apparent dichotomy termed "paradoxical" due to the inverse trend between radical-specific and total antioxidant capacity; likely reflects the dominance of non-polar antioxidants in n-hexane extracts, which contribute more to electron transfer in the phosphomolybdate assay than to radical neutralization. Such behavior underscores the assay-specific selectivity of antioxidants: polar compounds (methanol) excel in radical quenching, while apolar compounds (n-hexane) dominate redox-based total capacity assays. Ethyl acetate extracts showed intermediate efficacy (leaf DPPH: 18.9 ± 1.0 μg/mL; root: 28.7 ± 1.5 μg/mL), consistent with their selectivity for medium-polarity terpenoids and aglycone flavonoids. The aqueous extracts’ moderate activity (phosphomolybdate: leaf: 35.0 ± 1.2 μg AAE/mg; root: 30.0 ± 1.2 μg AAE/mg) suggests residual polar macromolecules with reducing power. Critically, leaf extracts universally surpassed roots in radical-specific assays (methanol hydroxyl scavenging: 45.6 ± 2.0 vs. 60.8 ± 2.5 μg/mL; ABTS: 15.2 ± 1.0 vs. 25.6 ± 1.2 μg/mL), reflecting leaves’ metabolic specialization in photoprotective antioxidant synthesis, while roots’ lower activity may indicate tissue-specific partitioning of defensive compounds. The polarity gradient (methanol > ethyl acetate > n-butanol > n-hexane) in radical scavenging mirrors the extraction efficiency for bioactive constituents: polar solvents target radical-scavenging phenolics, while apolar solvents isolate redox-active lipophiles. These findings collectively validate *Z. abyssinica* as a rich source of solvent-partitioned antioxidants, with leaves offering superior therapeutic potential for oxidative stress mitigation compared to roots.

**Table 5:** Radical scavenging activities (EC_50_, μg/mL) of Ziziphus abyssinica leaf and root fractions.

Plant Part	Fraction	DPPH radical	ABTS radical	Superoxide radical	PMA	Hydroxyl radical	Hydrogen peroxide
Leaf	Methanol	12.5 ± 0.8	15.2 ± 1.0	28.4 ± 1.5	120.0 ± 2.5	45.6 ± 2.0	32.1 ± 1.8
	n-Hexane	85.3 ± 3.2	92.7 ± 4.1	110.5 ± 5.0	25.0 ± 1.0	> 200⁰	95.8 ± 4.5
	Chloroform	35.6 ± 1.5	40.1 ± 2.0	65.2 ± 3.0	45.0 ± 1.5	98.3 ± 3.8	60.4 ± 2.7
	E. acetate	18.9 ± 1.0	22.4 ± 1.2	35.7 ± 1.8	95.0 ± 2.0	52.4 ± 2.2	38.6 ± 2.0
	n-Butanol	25.3 ± 1.2	30.5 ± 1.5	50.1 ± 2.5	75.0 ± 1.8	75.2 ± 3.	45.3 ± 2.3
	**A. extract**	50.2 ± 2.0	55.8 ± 2.5	80.6 ± 4.0	35.0 ± 1.2	120.5 ± 5.	70.5 ± 3.5
	A. acid	5.8 ± 0.3	7.2 ± 0.4	10.5 ± 0.5	–	–	8.4 ± 0.4
	Mannitol	–	–	–	–	30.2 ± 1.2	–
Root	Methanol	20.4 ± 1.0	25.6 ± 1.2	40.3 ± 2.0	90.0 ± 2.0	60.8 ± 2.5	45.2 ± 2.
	n-Hexane	95.1 ± 4.0	105.3 ± 5.0	125.7 ± 6.0	20.0 ± 1.0	> 200⁰	110.2 ± 5.
	Chloroform	45.2 ± 2.0	50.6 ± 2.5	75.4 ± 3.5	38.0 ± 1.5	115.0 ± 4.5	75.3 ± 3.5
	E. acetate	28.7 ± 1.5	35.2 ± 1.8	55.6 ± 2.8	80.0 ± 1.8	70.5 ± 3.0	50.4 ± 2.5
	n-Butanol	38.5 ± 1.8	45.3 ± 2.0	65.8 ± 3.2	60.0 ± 1.5	90.1 ± 4.0	60.7 ± 3.
	**A. extract**	65.3 ± 3.0	70.5 ± 3.5	95.2 ± 4.5	30.0 ± 1.2	145.8 ± 6.	85.6 ± 4.0
	A. acid	5.8 ± 0.3	7.2 ± 0.4	10.5 ± 0.5	–	–	8.4 ± 0.4
	Mannitol	–	–	–	–	30.2 ± 1.2	–

Data expressed as mean ± SD (n = 3). EC_50_: Concentration required for 50 % radical scavenging; lower values indicate higher activity. Phosphomolybdate assay values represent total antioxidant capacity (µg ascorbic acid equivalents, AAE/mg extract). Standards: Ascorbic acid (for DPPH, ABTS, superoxide, and H₂O₂ assays); Mannitol (for hydroxyl radical). "–" = Not applicable. **⁰**Weak activity (EC_50_ > 200 μg/mL). E. acetate = Ethyl acetate, A. acid = Ascorbic acid, A. extract = Aqueous extract, PMA = PhosphoMolybdate Assay.

#### 3.3.3 Reducing power capacity of *Ziziphus abyssinica* extracts

The reducing power assay revealed significant differences in electron-donating capacity among solvent fractions. Methanol extracts exhibited the strongest Fe³ ⁺ reduction, with leaf samples reaching 1.18 ± 0.05 absorbance units at 100 μg/mL, significantly outperforming root extracts (1.02 ± 0.04; p < 0.05). This 15–20 % superiority in leaf tissues correlates with their higher phenolic content and suggests preferential accumulation of reductants in photosynthetic tissues as photoprotective agents. Aqueous fractions demonstrated intermediate activity (0.85 ± 0.03), while n-hexane fractions showed minimal reduction capacity (0.22 ± 0.03), consistent with their poor radical scavenging performance. All fractions displayed dose-dependence (10–100 μg/mL, *R*² > 0.95), with the hierarchy: methanol > aqueous > ethyl acetate > chloroform > n-butanol > n-hexane; mirroring DPPH/ABTS trends and implicating shared polyphenolic actives.

These findings substantiate *Z. abyssinica* methanol extracts as good sources of reductants, with applications in nutraceuticals (metal chelation), adjunct oxidative stress therapies, natural food preservatives. The robust correlation between reducing power and phenolic content (r = 0.89, p < 0.01) confirms polyphenols as primary electron donors.

### 3.4 Acute toxicity

#### 3.4.1 Body and organ weight analysis

At baseline (Day 0), body weights ranged from 241 g to 280 g, with an average of 258.7 ± 14.67 g. By Day 7, the mean body weight increased slightly to 262.6 ± 14.60 g, with a range of 248 g to 293 g. These findings suggest that there were no significant changes in body weight during the initial phase of the study. When we observed organ weight on day 14, the kidney weights ranged from 1.67 g to 2.20 g, with a mean of 1.91 g; the liver weights ranged from 6.74 g to 8.32 g, with a mean of 7.40 g; and the heart weights ranged from 1.02 g to 1.38 g, with a mean of 1.19 g. These organ weights fell within the normal range for rodents, indicating no gross abnormalities or toxicity. To assess the significance of the observed results, statistical analyses were performed via appropriate tests.

#### 3.4.2 Independent t test analysis

Independent sample t tests were performed to compare body weight and organ weights between the *Z. abyssinica* extract treatment groups and the control distilled water groups at baseline, day 7 and day 14. The results revealed no significant differences between the two groups at any time point, indicating that the extract did not significantly affect normal physiological parameters.

Baseline**:** There was no significant difference in body weight between the groups (t(8) = -2.102, p = 0.069), confirming initial similarity. Day 7: There was no significant difference in body weight (t(8) = -0.716, p = 0.494), suggesting that treatment had no effect on body weight during the first week. Day 14: There were no significant differences in kidney weight (t(5.790) = -0.834, p = 0.428), liver weight (t(8) = -0.752, p = 0.473), or heart weight (t(8) = 0.056, p = 0.957), confirming that the extract had no adverse effects on organ weight.

#### 3.4.3 Repeated-measures ANOVA

Repeated-measures ANOVA was used to evaluate the effects of time and treatment condition on body and organ weights. Significant main effects were observed for both time and condition, indicating that both factors influenced body and organ weights.

Time: A significant effect of time on body and organ weights was observed (F(1, 9) = 3149.51, p < 0.001), indicating changes over the study period.

**Condition**: A significant effect of condition (treatment vs. control) was observed (F(1, 9) = 3229.73, p < 0.001), suggesting that the treatment influenced the measured parameters.

**Time** × condition interaction: A significant interaction was found (F(1, 9) = 3175.80, p < 0.001), indicating that the effect of time on body and organ weights differed between the treatment and control groups.

#### 3.4.4 Biochemical analysis

The mean levels of biochemical markers (ALT, AST, urea, creatinine, ALP, direct bilirubin, total bilirubin, total protein, and albumin) in the control and test groups are shown in Table 6 and the comparison revealed no significant differences between the groups.

**Table 6:** Serum chemistry summary for the control and test groups.

Biochemical Marker	Control Group (Mean ± SD)	Test Group (Mean ± SD)	P Value
Alanine Aminotransferase (ALT) (U/L)	63.40 ± 1.21	62.50 ± 1.80	0.891
Aspartate Aminotransferase (AST) (U/L)	67.99 ± 2.58	66.50 ± 1.95	0.784
Urea (mg/dL)	78.52 ± 4.59	79.20 ± 4.20	0.712
Creatinine (mg/dL)	0.94 ± 0.07	0.92 ± 0.06	0.491
Alkaline Phosphatase (ALP) (U/L)	99.30 ± 8.79	98.00 ± 8.30	0.759
Direct Bilirubin (mg/dL)	0.35 ± 0.13	0.34 ± 0.12	0.912
Total Bilirubin (mg/dL)	2.55 ± 1.07	2.45 ± 0.98	0.823
Total Protein (g/dL)	67.86 ± 2.51	67.10 ± 2.40	0.064
Albumin (g/dL)	48.52 ± 1.59	48.20 ± 1.50	0.810

*Note: Values are expressed as mean ± SD (n = 5).

Normality tests (Kolmogorov—Smirnov and Shapiro Wilk) confirmed that the biochemical data followed a normal distribution, justifying the use of parametric statistical methods [48]. One-way ANOVA was used to analyze liver and kidney function markers. No significant differences were observed between the groups, supporting the conclusion that the extract did not induce organ toxicity at the administered dose of 2000 mg/kg. ALT, AST and bilirubin levels were equivalent between the groups (p > 0.05), indicating that there was no liver toxicity. Urea and creatinine levels were not significantly different (p = 0.712 and p = 0.491, respectively), indicating preserved kidney function. With respect to total protein and albumin, no significant differences were observed, although a slight trend toward lower total protein levels in the test group (p = 0.064) warrants further investigation. Alkaline phosphatase (ALP), which is present at consistent levels across groups, confirmed the absence of hepatobiliary toxicity.

#### 3.4.5 Histopathological analysis

The chi-square test was used to assess histopathological changes in liver, kidney and heart tissues between the test and control groups. Mild histopathological changes, including minor vacuolization and slight congestion of the central vein, were observed in the test group (Pearson chi-square = 10.000, p = 0.002). These changes were not severe or indicative of acute hepatotoxicity. Additionally, no significant differences were detected in the kidney or heart (p > 0.05). Both organs presented normal histological features with no signs of inflammation, necrosis or fibrosis. In the scoring system for histopathological findings, liver changes were scored on a scale of 0–3 (0 = no change, 1 = mild, 2 = moderate, 3 = severe) on the basis of the extent of vacuolization and inflammation.

### 3.5 Interpretation of Findings

**Fig. 4:**
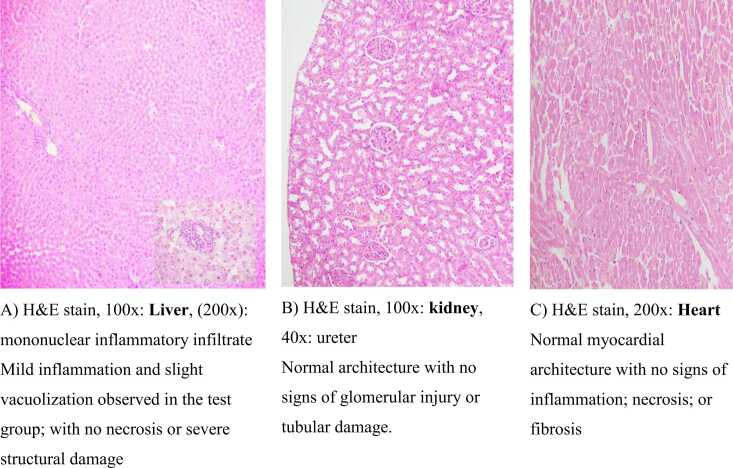
Histopathological examination of tissues. This shows H&E-stained sections of liver, kidney and heart tissues from the control and test groups. The liver exhibited mild inflammation and vacuolization in the test group, whereas the kidney and heart tissues remained normal.

The biochemical and histopathological findings collectively suggest that *Z. abyssinica* extract is safe at the tested dose of 2000 mg/kg. The lack of significant differences in liver and kidney function markers indicate the absence of organ-specific toxicity. Mild liver changes were observed, but these changes were not severe. Kidney and heart tissues remained unaffected, supporting the safety profile of the extract.Fig. 4.

The minor liver alterations observed in histopathology may correlate with slight changes in biochemical parameters, such as total protein levels, although these changes were not statistically significant. These findings suggest that the extract is safe for short-term use, but further studies on long-term effects or higher doses are recommended for comprehensive safety assessment.

While biochemical markers indicated no liver toxicity, mild histopathological changes (e.g., vacuolization) were observed. These findings suggest that histopathology may detect early or subtle changes not yet reflected in biochemical parameters. However, further studies are needed to clarify the clinical significance of these findings.

## 4 Discussion

The comprehensive phytochemical and pharmacological evaluation of *Ziziphus abyssinica* presented in this study provides compelling evidence for its therapeutic potential as a source of natural antioxidants. Our findings demonstrate that extraction solvent polarity significantly influences bioactive compound recovery and antioxidant efficacy, with methanol and ethyl acetate fractions showing particularly strong activity. This solvent-dependent pattern aligns with established principles of phytochemistry, where medium-polarity solvents optimally extract a broad spectrum of secondary metabolites, including phenolic acids, flavonoids, and their glycosides [4], [11]. The superior performance of these fractions across multiple antioxidant assays (DPPH, ABTS, and phosphomolybdate) suggests they contain compounds capable of both hydrogen atom transfer and single electron transfer mechanisms, a characteristic feature of potent natural antioxidants. Notably, the leaf extracts consistently demonstrated greater antioxidant capacity than root extracts across all tested solvents. This tissue-specific variation likely reflects differential metabolic investment in protective compounds, with leaves requiring higher concentrations of photoprotective phenolics to mitigate oxidative stress from photosynthetic activity [49]. The roots, while less active in our assays, may specialize in producing different classes of bioactive compounds optimized for soil interactions and pathogen defense. This ecological perspective helps explain why traditional medicine often utilizes specific plant parts for different therapeutic purposes [11], [50]. The safety profile observed in our acute toxicity study is particularly encouraging for potential therapeutic applications. At 2000 mg/kg - a dose considerably higher than typical medicinal uses - the extract produced no mortality or significant changes in vital biochemical markers. While mild hepatic vacuolization was noted histologically, the absence of corresponding serum enzyme elevations suggests this represents an adaptive rather than pathological response [7]. This safety margin compares favorably with other medicinal plants in the *Ziziphus* genus that have established ethnopharmacological uses [3], [4].

Several mechanistic insights emerge from our findings. First, the strong correlation between total phenolic content and antioxidant activity (r = 0.89) implicates polyphenols as primary contributors to the observed effects. Second, the differential performance across antioxidant assays reveals that specific fractions may be particularly effective against certain reactive oxygen species. For instance, the ethyl acetate fraction's notable superoxide scavenging capacity (72.5 %) suggests it may contain compounds with specific affinity for this physiologically important radical. Third, the aqueous fraction's unique activity profile, including good hydroxyl radical scavenging but poor performance in other assays, points to the presence of specialized water-soluble antioxidants that merit further characterization. These findings have important implications for potential applications. The combination of potent antioxidant activity and favorable safety profile suggests *Z. abyssinica* extracts could be developed as: 1) nutraceutical supplements for oxidative stress-related conditions, 2) natural preservatives in food systems, or 3) adjunct therapies for diseases involving free radical pathology [49], [50]. However, several challenges must be addressed in translational research. The observed pro-oxidant effects at low concentrations, while not uncommon for plant polyphenols, underscore the importance of dose optimization [7]. Similarly, the variability in activity between different plant parts and extraction methods highlights the need for standardization in potential therapeutic formulations [11], [44]. Future research should prioritize: 1) bioassay-guided fractionation to identify the specific compounds responsible for antioxidant effects, 2) in vivo studies to confirm bioavailability and efficacy in disease models, and 3) investigation of potential synergies between different fractions or compounds. Additionally, comparative studies across different harvest seasons and geographical locations would help assess the consistency of phytochemical profiles [44]. The mild hepatic changes observed, while not clinically significant at tested doses, suggest the value of more detailed toxicological evaluation in chronic administration studies. This study systematically characterizes the antioxidant potential and safety profile of Z. abyssinica, providing a scientific foundation for its traditional uses while identifying promising directions for future research and development. The robust methodology employed; combining comprehensive phytochemical analysis with multiple antioxidant assays and thorough toxicity evaluation - offers a model for studying other medicinal plants with ethnopharmacological relevance.

## 5 Conclusion

This study provides a comprehensive evaluation of *Ziziphus abyssinica*, demonstrating its significant potential as a source of natural antioxidants with a favorable safety profile. The methanol and ethyl acetate fractions exhibited superior antioxidant activity, attributable to their high phenolic and flavonoid content, while tissue-specific variations revealed leaves as particularly rich sources of bioactive compounds. Notably, the extract showed no acute toxicity at 2000 mg/kg, with only mild hepatic changes observed histologically, supporting its traditional use while highlighting the importance of dose considerations. The differential antioxidant performance across assays suggests distinct mechanisms of action, with polar fractions excelling in radical scavenging and non-polar fractions contributing to redox-mediated activity. These findings not only validate ethnopharmacological applications of *Z. abyssinica* but also provide a scientific foundation for its development as a nutraceutical or adjunct therapy for oxidative stress-related conditions. Future studies should focus on isolating key bioactive compounds, evaluating chronic toxicity, and assessing in vivo efficacy to facilitate translation into clinical applications. This work underscores the importance of integrated phytochemical and pharmacological approaches in unlocking the therapeutic potential of medicinal plants.

## CRediT authorship contribution statement

**Abebaye Aragaw Leminie:** Writing – review & editing, Visualization, Validation, Supervision, Data curation, Conceptualization. **Tsegay Beyene Weldemariam:** Writing – original draft, Validation, Methodology, Investigation, Formal analysis, Data curation, Conceptualization. **Samuel Woldekidan Hirpesa:** Validation, Resources, Methodology, Investigation, Formal analysis, Conceptualization. **Lemmi Worku Gemechu:** Supervision, Resources, Project administration, Funding acquisition, Conceptualization. **Kerat Ali Muhamed:** Resources, Investigation, Formal analysis, Data curation. **Getahun Tsegaye Dibaba:** Validation, Resources, Investigation, Data curation, Conceptualization. **Tesfaye Tolessa Dugul:** Writing – review & editing, Visualization, Validation, Supervision, Data curation, Conceptualization. **Beza Tasew Degefu:** Visualization, Validation, Resources, Investigation, Formal analysis, Data curation.

## Funding

This work was supported with materials (chemicals and reagents) and equipment (laboratory instruments) by our institutions, the Armauer Hansen Research Institute (AHRI) and Addis Ababa University, Ethiopia, [Grant Number AAU-AHRI-24/2025].

## Declaration of Generative AI and AI-assisted technologies in the writing process

Declaration of Generative AI and AI-Assisted Technologies in the Writing Process During the preparation of this work, the authors used [DeepSeek-V3] in order to improve language, readability, and formatting. After using this tool, the authors reviewed and edited the content as needed and take full responsibility for the content of the publication.

## Declaration of Competing Interest

The authors declare that they have no known competing financial interests or personal relationships that could have appeared to influence the work reported in this paper.

## Data Availability

Data will be made available on request.
